# Left atrial concomitant surgical ablation for treatment of atrial fibrillation in cardiac surgery: A meta-analysis of randomized controlled trials

**DOI:** 10.1371/journal.pone.0191354

**Published:** 2018-01-23

**Authors:** Xinxin Wang, Chunguo Wang, Minhua Ye, Jiang Lin, Jiang Jin, Quanteng Hu, Chengchu Zhu, Baofu Chen

**Affiliations:** Department of Thoracic and Cardiovascular Surgery, Affiliated Taizhou Hospital of Wenzhou Medical University, Taizhou, China; University of Milano, ITALY

## Abstract

**Introduction:**

Surgical ablation is a generally established treatment for patients with atrial fibrillation undergoing concomitant cardiac surgery. Left atrial (LA) lesion set for ablation is a simplified procedure suggested to reduce the surgery time and morbidity after procedure. The present meta-analysis aims to explore the outcomes of left atrial lesion set versus no ablative treatment in patients with AF undergoing cardiac surgery.

**Methods:**

A literature research was performed in six database from their inception to July 2017, identifying all relevant randomized controlled trials (RCTs) comparing left atrial lesion set versus no ablative treatment in AF patient undergoing cardiac surgery. Data were extracted and analyzed according to predefined clinical endpoints.

**Results:**

Eleven relevant RCTs were included for analysis in the present study. The prevalence of sinus rhythm in ablation group was significantly higher at discharge, 6-month and 1-year follow-up period. The morbidity including 30 day mortality, late all-cause mortality, reoperation for bleeding, permanent pacemaker implantation and neurological events were of no significant difference between two groups.

**Conclusions:**

The result of our meta-analysis demonstrates that left atrial lesion set is an effective and safe surgical ablation strategy for AF patients undergoing concomitant cardiac surgery.

## Introduction

Atrial fibrillation (AF) is the most common cardiac arrhythmia in clinical practice and associated with reduced survival and increased risk of stroke, with a prevalence of 30 to 50% in patients undergoing mitral valve surgery[1–6]. The pathology of AF is supposed to be abnormal re-entry circuits existing in atrial walls. The first surgical procedure called Cox-Maze III procedure was introduced by Dr James L Cox in 1992[7]. The Cox-Maze III operation is a complex procedure which involves a series of endocardial incisions in both atria. The aim of Cox-Maze procedure is to interrupt the multiple, disorganized re-entrant circuits[8]. This procedure is usually performed with other cardiac surgery including mitral valve repair/replacement or coronary artery bypass graft. Recent researches renewed the pathophysiological mechanisms underlying AF and found the pulmonary veins and left atrium were the main “triggers” of AF[9]. This discovery lead to the innovation of the traditional Cox-Maze III procedure and limited the incisions to left atrium even just pulmonary veins instead of both atria. On the other hand, the advent of new energy sources for maze operation including radiofrequency, cryo-energy and microwaves also reduced the complex of the procedure. Previous meta-analysis has demonstrated that surgical ablation is an effective and safe procedure for AF patients undergoing cardiac surgery or mitral valve surgery only[10, 11]. However, the direct evidence respecting to outcomes of left atrial surgical ablation for AF patients were not established. In order to make a supplement to this field, the present meta-analysis aims to summarize the available randomized evidence about the clinical outcomes of left atrial surgical ablation in patients undergoing concomitant surgery.

## Materials and methods

### Literature search strategy

Electronic searches were performed using PubMed, Ovid Medline, Cochrane Central Register of Controlled Trials (CCTR), Cochrane Database of Systematic Reviews (CDSR), Database of Abstracts of Review of Effectiveness (DARE) and ACP Journal Club from their date of inception to July 2017. To achieve the maximum sensitivity of the search strategy, we combined the terms: ‘atrial fibrillation’ AND ‘ablation’ AND ‘randomized controlled trial’ as either key words or medical subject headings (MeSH) terms. The reference lists of all retrieved articles were checked for further identification of extra relevant studies assessed using the inclusion and exclusion criteria.

### Selection criteria

Inclusion criteria for the present systematic review and meta-analysis were as follows:

1. Randomized controlled trial.
2. Patients underwent any cardiac surgery concomitantly with surgical ablative treatment of atrial fibrillation.
3. All patients were diagnosed with permanent or persistent atrial fibrillation.
4. Surgery ablation techniques included the left atrial surgical ablation.
5. A direct comparison between cardiac surgery with or without left atrial surgical ablation.
6. The endpoints of study were sinus rhythm or AF-free survival.

Exclusion criteria for the present systematic review and meta-analysis were as follows:

1. Not a randomized controlled trial.
2. Catheter ablation without concomitant cardiac surgery.
3. Comparison between other ablation techniques or different ablation energy, such as Cox-maze, modified Cox-maze, biatrial surgical ablation, cut and sew, cryoablation, radiofrequency, mircrowave, bipolar and unipolar.
4. Patients with paroxysmal AF were included.
5. Duplicate data from the same study.

### Data extraction and quality assessment

All data were extracted from article texts, tables and figures. Two investigators independently reviewed every retrieved article (XW and QH). A disagreement was solved by discussion and consensus with a third investigator (CW) if necessary. The risk of bias was assessed according to the Cochrane Collaboration for risk of bias, by two reviewers (JL and JJ). The final results were reviewed by senior investigators (MY and BC).

### Statistical analysis

Clinical outcomes were assessed with a standard meta-analysis technique. The hazard ratio (HR) and odds ratio (OR) was used as a summary statistic. χ^2^ tests were used to examine heterogeneity between trials. I^2^ statistic was used to estimate the heterogeneity and I^2^>50% was considered as substantial heterogeneity. In the present meta-analysis, the results were analyzed with the random-effects model considering the possible clinical diversity and methodological variation between studies. HR and the corresponding 95% CI were used for freedom from atrial fibrillation indirectly calculated using the method of Tierney and colleagues[12] in each study. If there was a substantial heterogeneity, the possible reasons for this were explored qualitatively. Meta-regression was used to investigate the effects of covariates, especially variations in patient characteristics. Publication bias of the major outcomes of this meta-analysis was detected by Egger’s regression test. All P values were two-sided. All statistical analyses were conducted with Review Manager version 5.3 (The Cochrane Collaboration, Copenhagen, Denmark) and Stata (version 12.0; StataCorp, College Station, TX).

## Results

### Literature search

**Fig 1:**
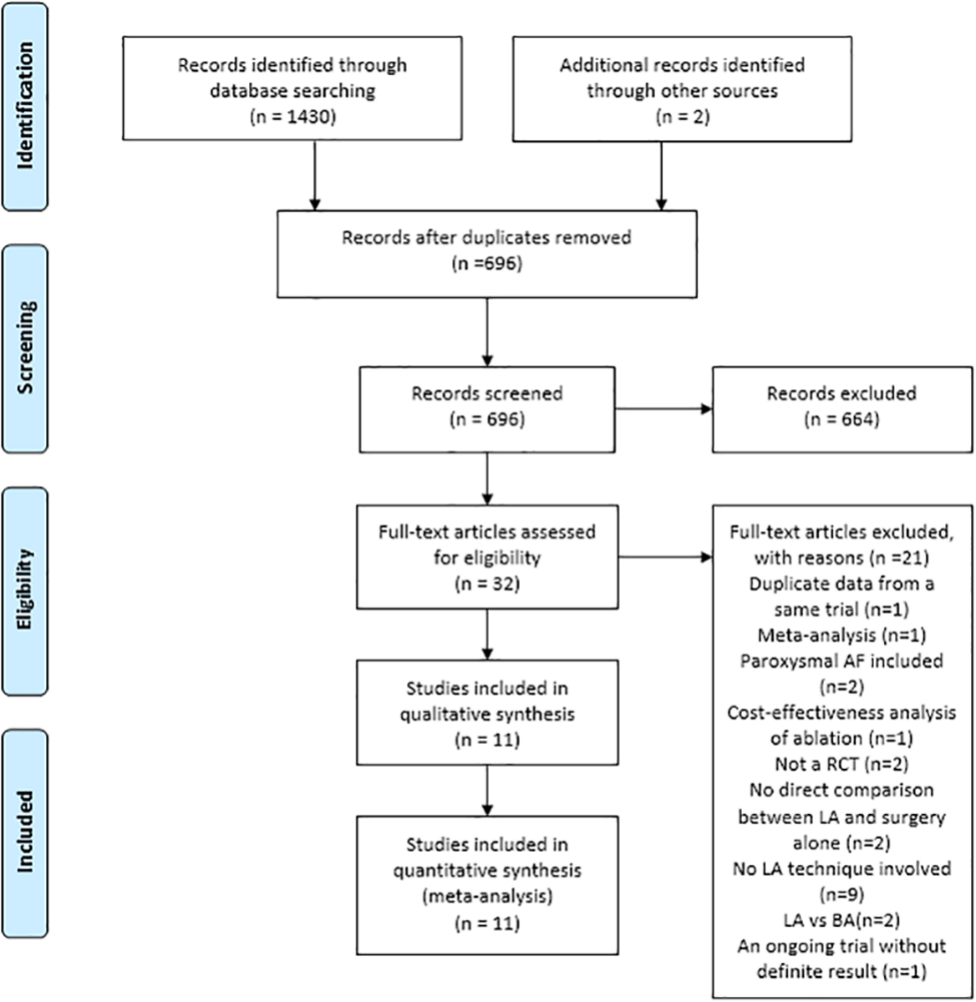
Search strategy of meta-analysis for left atrial surgical ablation with cardiac surgery (CS + LA) versus cardiac surgery (CS) alone in atrial fibrillation (AF) patients.

A total of 1430 references were identified through six electronic database searches. Manual search of reference lists yielded two extra studies. After exclusion of duplicate or irrelevant references, 696 potentially relevant articles were retrieved. After detailed evaluation of these articles, 32 studies remained for assessment. After applying the selection criteria, 11 RCTs were selected for analysis (Fig 1). In these eleven studies, 666 patients underwent procedures that involved cardiac surgery with left atrial ablation (CS + LA group; n = 333) or without surgical ablation (CS group; n = 333). The study characteristics of these trials are summarized in Table 1.

**Table 1:** Summary of RCTs comparing CS+LA versus CS surgical treatment in patients with AF/.

First author (reference)	Year	Institution	Study period	CS+LA	CS	Energy	CS type	Primary endpoint	Monitoring	AAD indication during follow up
Wang	2014	Fuwai Hospital (Beijing, China)	2008–2011	70	70	Cut-and-sew	CABG, MV, TV, AV	Sinus rhythm	ECG, 24 h Holter, Echo	Amiodarone maintained for 3 months to all patients, then β-blockers administrated for recurrent AF
Vasconcelos	2004	Instituto do Coracao (São Paulo, Brazil)	2000–2002	15	14	Cut-and-sew	MV, TV	AF-free survival	ECG, Echo	Amiodarone administered for postoperative AF until discharge
Srivastava	2008	King Edward Memorial Hospital (Mumbai, India)	2000–2005	40	40	CY	MV, TV	Sinus rhythm	ECG, 2D echo	Amiodarone for 2 months to patients still in AF after procedure and DC conversion
Schuetz	2003	Ludwig-Maximilians University (Munich, Germany)	2001–2002	24	19	MW	CABG, MV, TV, AV	Sinus rhythm	ECG, 24 h Holter	Amiodarone or sotalol given to patients with SR restored and no contraindications
Knaut	2010	University of Technology (Dresden, Germany)	NR	24	21	MW	CABG, AV	Sinus rhythm	ECG, 24 h Holter, cardioversion	β-blocker to stabilize SR, cardio version for recurrent AF in 90 postoperative days
Doukas	2005	Glenfield Hospital (Leicester, England)	2001–2003	49	48	Cut-and-sew	MV, CABG, TV	Sinus rhythm	ECG, 24 h Holter, cardioversion	Amiodarone or sotalol for at least 3 months to all patients, then withdrawn. Various antiarrhythmic agents for patients still in AF after 3 months
De Lima	2004	Fundação Universitária de Cardiologia (Porto Alegre, Brazil)	1999–2004	10	10	Cut-and-sew	MV	Sinus rhythm	24 h ECG, Echo	Amiodarone or sotalol given to control rhythm without specific information
Chevalier	2009	Hopital Louis Pradel (Louis-Pradel, France)	2002–2005	21	22	RF	MV, TV, AV	Sinus rhythm	Holter, Echo	Not mentioned
Cherniavsky	2014	Novosibirsk Research Institute of Circulation Pathology, (Novosibirsk, Russia)	2008–2011	30	34	RF	CABG	AF-free survival	ILR, Cardiac Compass	Amiodarone administrated for 3 months to all patients
Blomstrom- Lundqvist	2007	Uppsala University Hospital (Uppsala, Sweden)	2003–2005	30	35	CY	MV, CABG, TV	Sinus rhythm	Cardiac telemetry, ECG, echo, cardioversion	Amiodarone or sotolol given for postoperative AF. Prophylactic antiarrhythmic drugs for 3 months to patients with postoperative AF that required cardioversion
Albrecht	2009	Fundação Universitária de Cardiologia (Porto Alegre, Brazil)	1999–2004	20	20	Cut-and-sew	MV	Sinus rhythm	ECG, echo, treadmill stress test	Amiodarone given to patients who had cardioversion to maintain SR

AF, atrial fibrillation; AV, aortic valve surgery; CABG, coronary artery bypass grafting; CS: cardiac surgery; CY, cryoablation; echo, echocardiography; LA, left atrial; MV, mitral valve surgery; MW, microwave; NR, not reported; RCT, randomised controlled trial; RF, radiofrequency; TV, tricuspid valve surgery; ILR implantable loop recorder.

### Quality assessment

**Fig 2:**
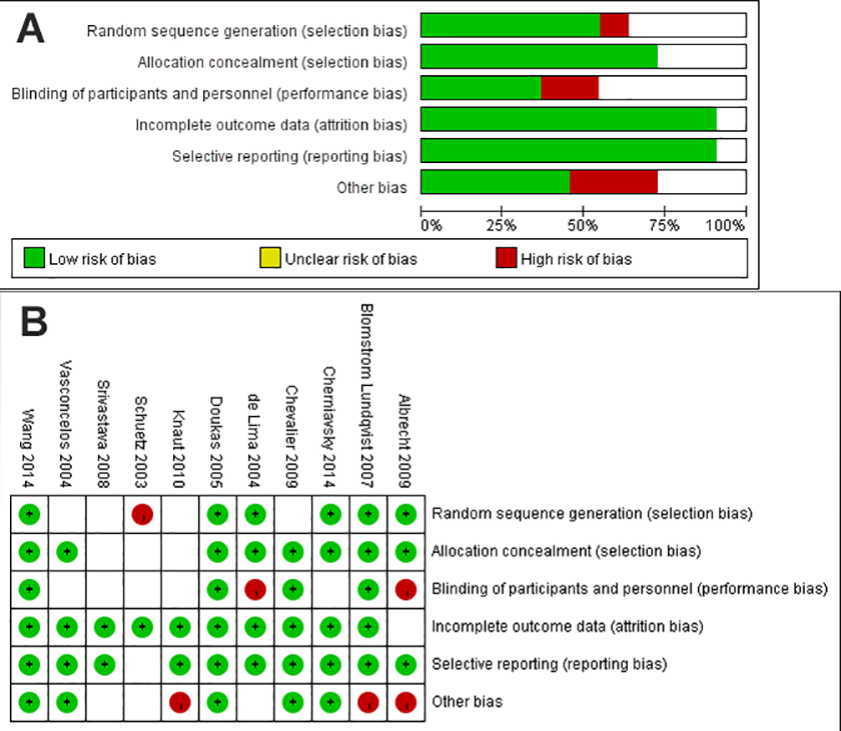
(A) Methodological quality graph and (B) Methodological quality summary for the risk of bias from randomized controlled trials comparing CS+LA versus CS alone for treatment of AF. Blank boxes represent unclear risk of bias, due to inadequate methodological descriptions from the original publication.

These eleven studies were all RCTs[13–23]. One study had more than 50 patients[13], while the remaining ten studies had less than 50 patients (range, 10–49 patients). Five studies used cut-and-sew[13, 14, 18, 19, 23], two studies used radiofrequency ablation[20, 21], two studies used cryoablation[15, 22] and two studies used microwave[16, 17]. All studies reported patients undergoing left atrial ablation and left appendage amputation. A little variant existed among studies with regarding to ablation or cut-and-sew lines on the left atrial wall. All studies except one[17] reported a line connecting pulmonary veins and mitral valve annulus. Eight studies made a line connecting pulmonary veins and appendage amputation site[13–18, 21, 22]. Six studies isolated the pulmonary veins in one circle[14–17, 19, 23]. Four studies isolated the left and right pulmonary veins in pair[13, 18, 21, 22]. One study isolated the pulmonary veins in pair or one by one[20]. The five studies which isolated the pulmonary veins in pair or one by one made a connection line between two pairs. Only two studies reported performing cryoablation or electrocauterization to coronary sinus[15, 23]. The lesion set description of included studies were summarized in Table 2. All studies focused on permanent or persistent AF. SR was the primary endpoint in nine studies, while AF-free survival was the primary endpoint in two studies. The data of SR at discharge was reported in 8 of 11 studies. Preoperative data for co-variates, which were available for both groups, were variable between the studies in Table 3. Out of the 8 studies, age and gender were reported in 8, LVEF in 7, LAD in 8, AF duration in 7, NYHA III/IV in 2, hypertension in 4, stroke and diabetes in 3. As such, the NYHA, stroke and diabetes were excluded from meta-regression analysis for sinus rhythm at discharge. The data of freedom from atrial fibrillation were extracted indirectly from 7 of 11 studies. Out of the 7 studies, age, gender, LVEF and LAD were reported in 7, AF duration in 6, NYHA III/IV, hypertension, stroke and diabetes in 3. As such, the NYHA, stroke and diabetes were excluded from meta-regression analysis for freedom from atrial fibrillation at 1 year follow up. 30-day mortality was reported by all eleven studies. The 11 RCTs were also assessed qualitatively using tools recommended by the Cochrane Collaboration for the risk of bias. A graph and summary of selection bias, performance bias, detection bias, attrition bias, reporting bias and other bias identified in each individual RCT is shown in Fig 2.

**Table 2:** Lesion set description of included studies.

First author	PVI	Interconnecting PV	LAA	PV-MV annulus	PV-LAA	Mitral CS
Wang	Yes^a^	Yes	Yes	Yes	Yes	No
Vasconcelos	Yes^b^	No^b^	Yes	Yes	Yes	No
Srivastava	Yes^b^	No^b^	Yes	Yes	Yes	Yes
Schuetz	Yes^b^	No^b^	Yes	Yes	Yes	No
Knaut	Yes^b^	No^b^	Yes	No	Yes	No
Doukas	Yes^a^	Yes	Yes	Yes	Yes	No
De Lima	Yes^b^	No^b^	Yes	Yes	No	No
Chevalier	Yes^a^	Yes	Yes^c^	Yes	No	No
Cherniavsky	Yes^a^	Yes	Yes	Yes	Yes	No
Blomstrom-Lundqvist	Yes^a^	Yes^a^	Yes	Yes	Yes	No
Albrecht	Yes^b^	No^b^	Yes	Yes	No	Yes

PVI: pulmonary vein isolation; LAA: left atrial appendage; EMW: epicardial microwave ablation; MM: modified maze; MV: mitral valve; CS: coronary sinus.

^a^PVI performed one-by-one or pair-by-pair, an interconnecting line was performed between left and right pairs of PVs

^b^Encircling lesion around all four PVs performed (not one-by-one or pair-by-pair)

^c^Surgeon’s preference; cardiopulmonary bypass duration.

**Table 3:** Summary of baseline patient characteristics and risk factors in studies comparing CS+LA with CS alone in surgical treatment for atrial fibrillation.

First author	Age		Male (%)		LVEF (%)		LAD (mm)		AF duration (mo)		NYHA III-IV (%)		Hypertension (%)		Prior stroke (%)		Diabetes (%)	
	CS+LA	CS	CS+LA	CS	CS+LA	CS	CS+LA	CS	CS+LA	CS	CS+LA	CS	CS+LA	CS	CS+LA	CS	CS+LA	CS
Wang	52±10	54±10	40	41	61±7	61±6	54±7	51±9	35±21	34±21	43	40	7	6	11	11	3	6
Vasconcelos	50±10	51±10	27	43	69±9	66±11	55±5	55±5	24±20	34±29	NR	NR	NR	NR	NR	NR	NR	NR
Srivastava	36±8	37±10	55	58	NR	NR	50±7	49±6	12	12	NR	NR	NR	NR	NR	NR	NR	NR
Schuetz	65±10	70±8	50	74	63±13	54±17	55±11	54±18	46±34	111±111	NR	NR	NR	NR	NR	NR	NR	NR
Knaut	74±4	75±6	58	67	56±14	54±6	45±4	47±6	71±53	52±96	NR	NR	83.3	90.5	4.1	4.7	66.6	47.6
Doukas	67±9	67±8	63	50	57±6	58±7	58±7	60±11	57±55	47±64	NR	NR	30.6	22.9	NR	NR	2.0	4.2
De Lima	54±9	50±15	30	60	64±12	64±10	53±9	62±12	23^M^	17^M^	70	80	NR	NR	NR	NR	NR	NR
Chevalier	70±6	66±10	24	50	60±9	61±9	55±11	53±11	161	89.2	NR	NR	66	50	0	13	NR	NR
Cherniavsky	62±7	64±8	83	74	56±14	53±11	49×55	49×50	NR	NR	NR	NR	53	50	10	24	10	24
Blomstrom- Lundqvist	70±8	66±8	83	83	54±9	57±12	61±11	58±7	26±33	33±54	66.7	68.6	30	31.4	3.3	8.6	6.7	6.7
Albrecht	55±9	51±15	30	50	62±11	63±7	53±8	62±12	32±32	25±32	70	80	NR	NR	NR	NR	NR	NR
Minimum	36	37	24	41	54	53	45	47	12	12	43	40	7	6	0	4.7	2	4.2
Maximum	74	75	83	83	69	66	61	62	161	111	70	80	83.3	90.5	11	24	66.6	47.6
Weighted average	59	59	52	57	59	59	54	54	46	42	55	56	35	32	7	13	12	13
P	0.71		0.22		0.33		0.26		0.85		0.24		0.85		0.09		0.99	

CS: cardiac surgery; LA: left atrial ablation; LVEF, left ventricular ejection fraction; LAD, left atrial diameter; AF, atrial fibrillation; NYHA, New York Heart Association Functional Classification; NR, not reported; M, median

### Baseline patient and operational characteristics

The baseline patient and operational characteristics are summarized in Table 3. Similar baseline characteristics were observed in both groups. Males accounted for 24–83% of patients undergoing CS + LA and 41–83% undergoing CS alone (weighted mean: 52% vs. 57%; P > 0.05). The average age ranged between 35–75 years for both CS + LA and CS groups (weighted mean: 59 vs. 59; P = 0.71) for CS + LA and CS groups respectively. There were also no differences between CS+LA and CS groups in terms of LVEF (P = 0.33), LA diameter (LAD) (P = 0.26), NYHA III/IV (P = 0.24), prior stroke (P = 0.09) and diabetes (P = 0.99). CBP and aortic cross clamp time was significantly longer when cardiac surgery was performed concomitantly with left atrial surgical ablation (P <0.01). Five studies reported valvular surgery with surgical ablation and one study reported CABG with surgical ablation. Three studies reported mixed valvular and CABG surgery. Two studies didn’t give specific information about surgery type. The operational characteristics are summarized in Table 4.

**Table 4:** Summary of perioperative characteristics and complications.

First author	CBP time (min)		Cross-clamp time (min)		CABG (%)		Valvular surgery (%)		30-day mortality (%)		Reoperation for bleeding (%)	
	CS+LA	CS	CS+LA	CS	CS+LA	CS	CS+LA	CS	CS+LA	CS	CS+LA	CS
Wang	101.0 ± 34.0	85.3 ±34.7	72.1 ± 28.3	61.9 ± 29.3	NR	NR	100	100	0	0	NR	NR
Vasconcelos	106±17	78±24	NR	NR	0	0	100	100	6.7	0	6.7	0
Srivastava	NR	NR	NR	NR	0	0	100	100	0	0	5	5
Schuetz	121±27	104±45	100±25	74±44	12.5	26	66.7	36.8	4.2	5.3	NR	NR
Knaut	NR	NR	NR	NR	54	57	NR	NR	8.3	0	NR	NR
Doukas	106±34	99±37	70±26	64±28	10.2	12.5	100	100	6.1	8.3	NR	NR
De Lima	97.8±3	68.3±22	NR	49.1±19	0	0	100	100	0	0	0	10
Chevalier	NR	NR	93±32	74±19	0	0	100	100	4.8	0	NR	NR
Cherniavsky	105.2 ± 37.2	70.8 ± 40.6	73.1 ± 28.2	47.5 ± 32.9	100	100	0	0	0	0	0	0
Blomstrom- Lundqvist	147±23	119±33	87±95	84±23	20	14.3	100	100	3.3	0	5.9	5.7
Albrecht	99.85± 23.8	62.0 ± 23.8	74.7 ± 19.2	45.10 ± 21.1	0	0	100	100	5	0	0	0
Minimum	97.8	62	70	45.1	0	0	0	0	0	0	0	0
Maximum	147	119	100	84	100	100	100	100	8.3	8.3	6.7	10
Weighted average	110.1	89.1	78.4	64.1	21.7	23.5	87.7	85.2	3	1.5	3.3	3.3
P	0.0003		0.006		0.93		0.85		0.09		0.82	

CS: cardiac surgery; LA: left atrial ablation; CBP, cardiopulmonary bypass time; NR, not reported; CABG, coronary artery bypass grafting

### Assessment of efficacy

#### Prevalence of sinus rhythm and freedom from atrial fibrillation

**Fig 3:**
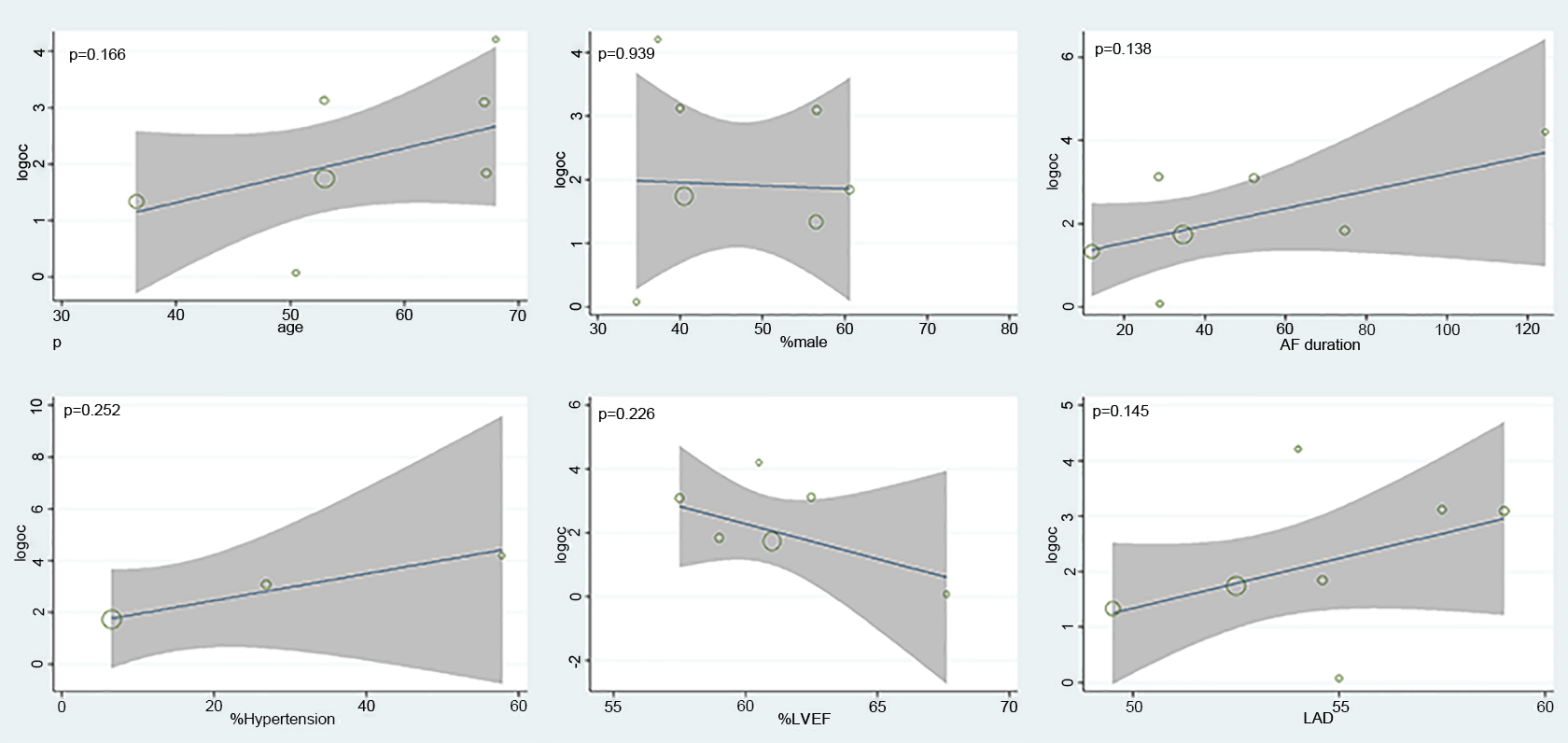
Forest plot of sinus rhythm prevalence at discharge, 6-month, and 1 year follow-up, showing summary of ORs with 95% confidence intervals for included studies.

**Fig 4:**
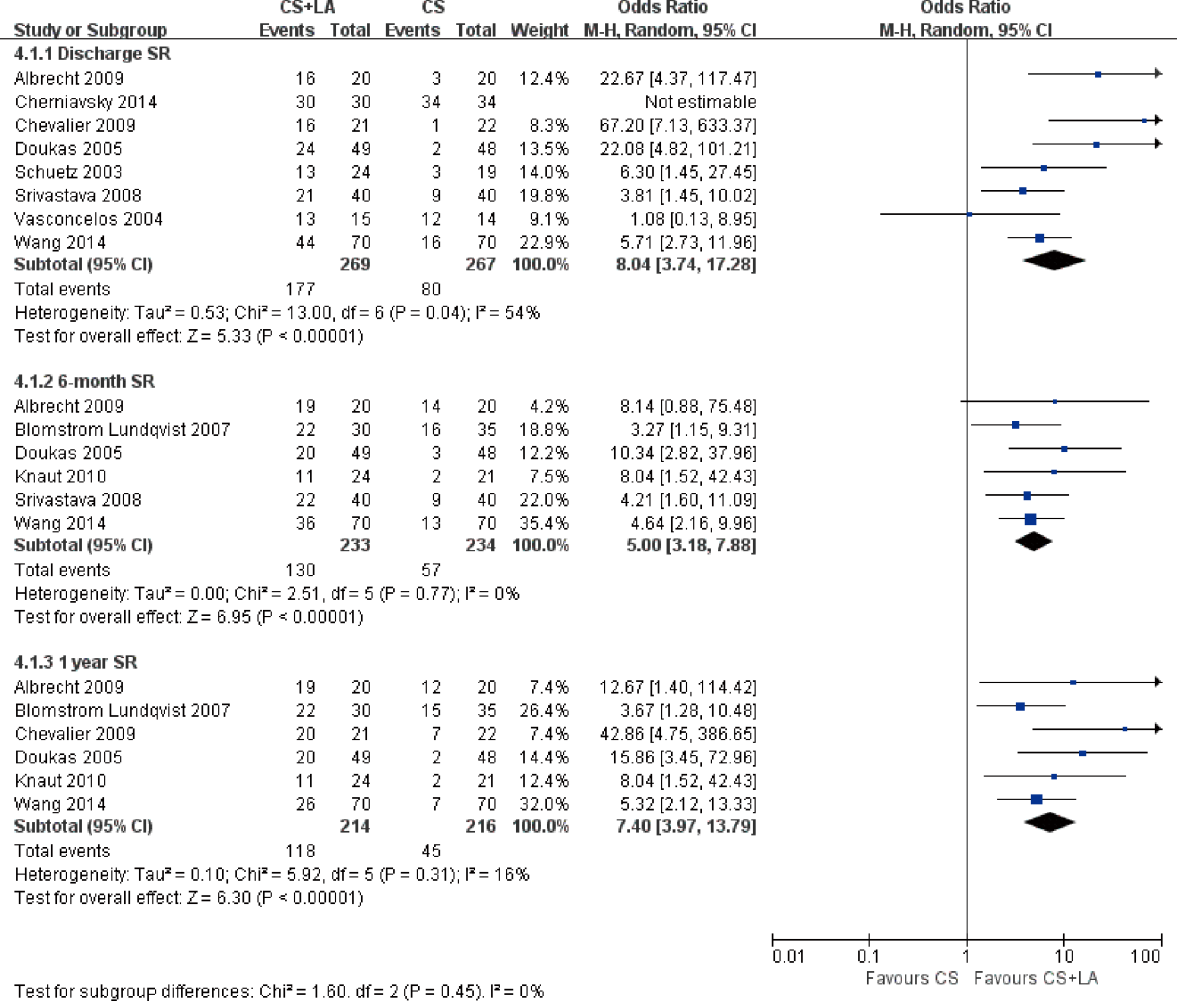
Meta-regression analysis assessing the effect of various patient characteristics on sinus rhythm at discharge. LVEF: left ventricular ejection fraction; LAD: left atrial diameter.

**Fig 5:**
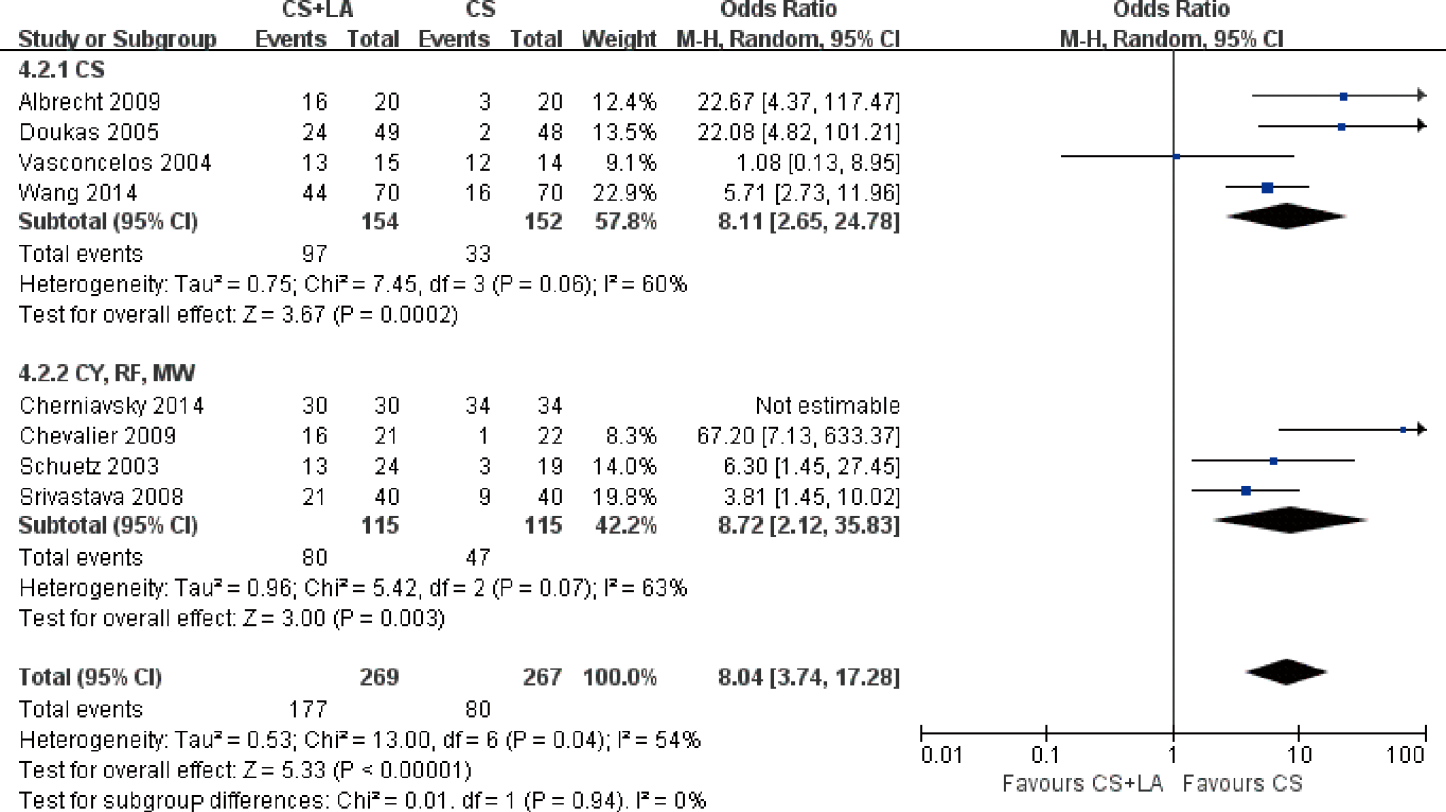
Forest plot of subgroup analysis for sinus rhythm prevalence at discharge according to ablation energy source, showing summary of ORs with 95% confidence intervals for included studies.

**Fig 6:**
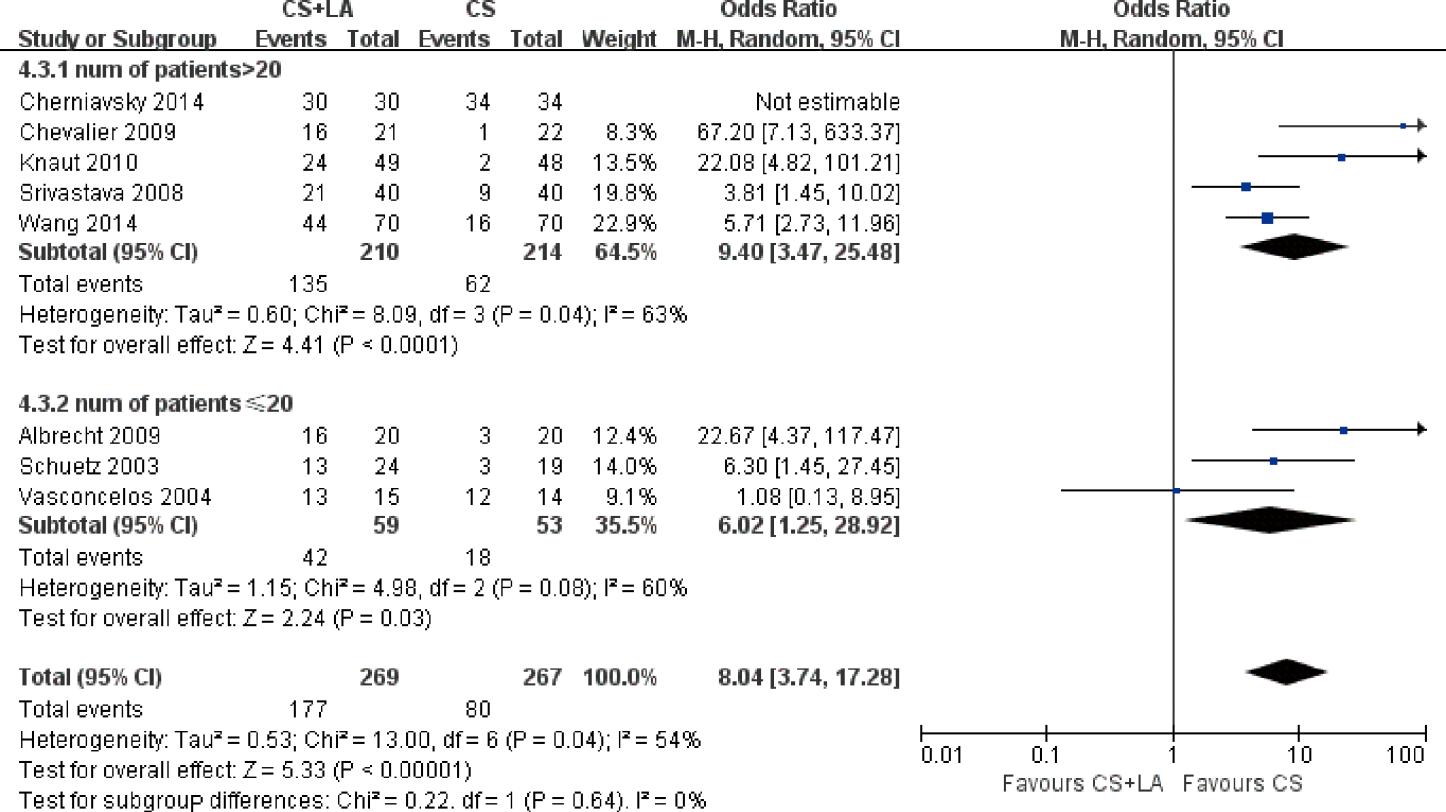
Forest plot of subgroup analysis for sinus rhythm prevalence at discharge according to number of patients, showing summary of ORs with 95% confidence intervals for included studies.

**Fig 7:**
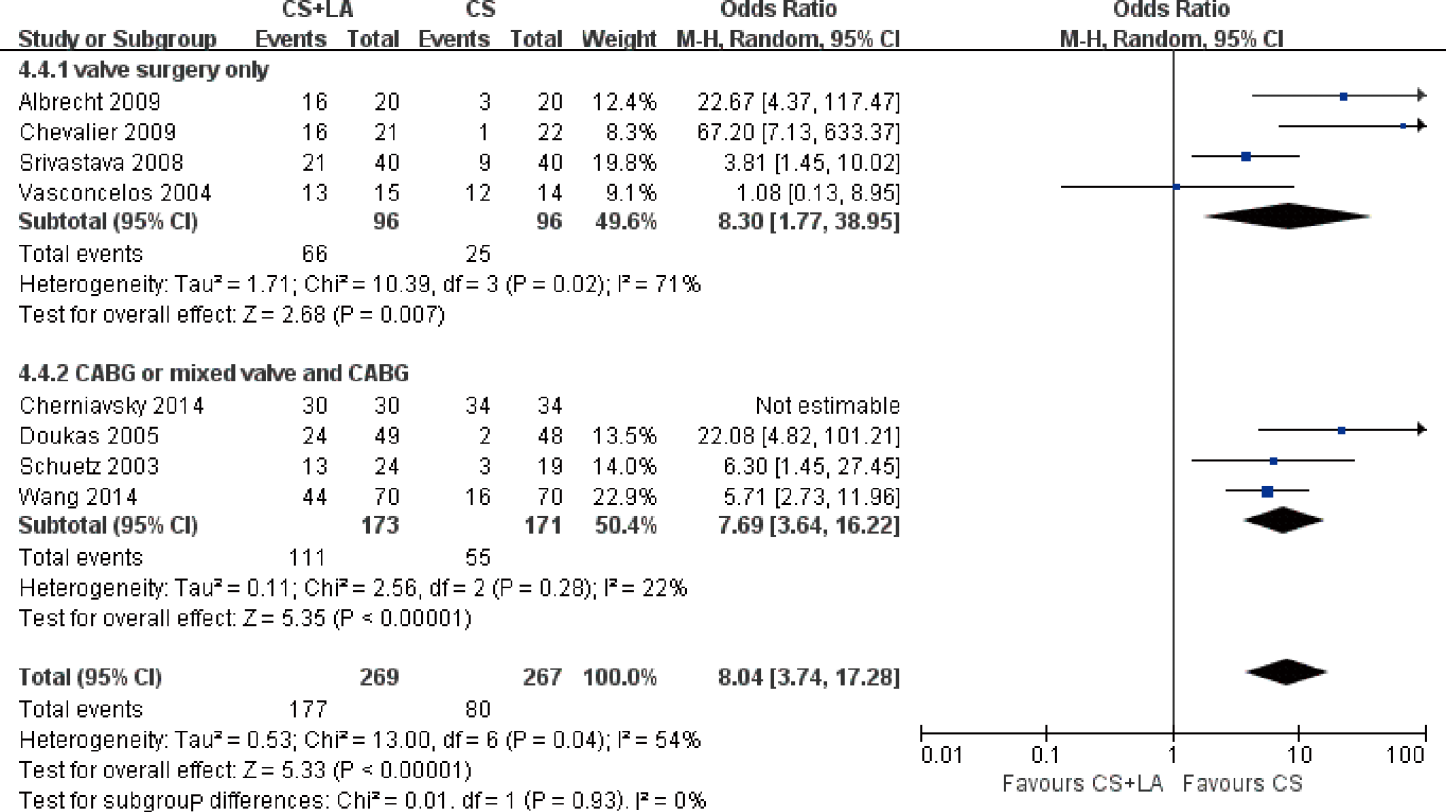
Meta-regression analysis assessing the effect of various patient characteristics on freedom for atrial fibrillation at 1 year follow up. LVEF: left ventricular ejection fraction; LAD: left atrial diameter.

**Fig 8:**
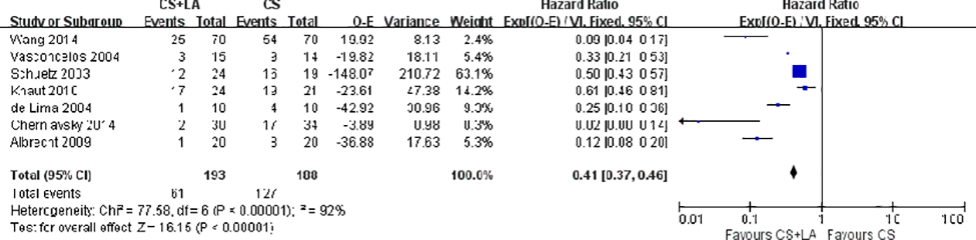
Forest plot of 1-year freedom from atrial fibrillation at 1 year follow-up, showing summary of HRs with 95% confidence intervals for included studies.

**Fig 9:**
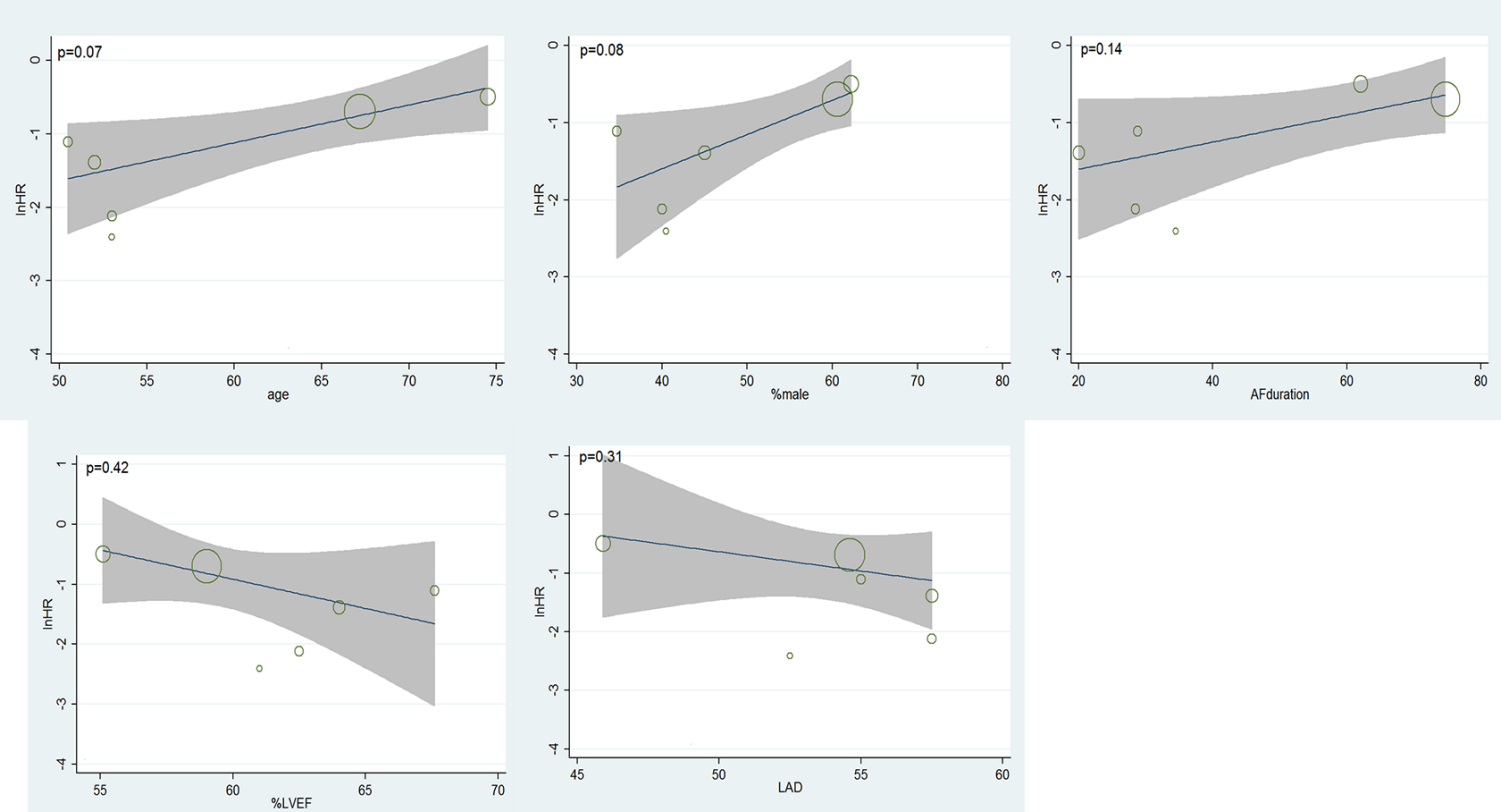
Meta-regression analysis assessing the effect of various patient characteristics on freedom for atrial fibrillation at 1 year follow up. LVEF: left ventricular ejection fraction; LAD: left atrial diameter.

The number of patients in SR at discharge was significantly higher in the CS + LA group compared to the CS group (65.8% vs. 30.0%; OR, 8.04; 95% CI, 3.74–17.28; P<0.00001; I^2^ = 54%). The CS + LA group also had a significantly higher proportion of patients in SR compared to CS group at 6-month (55.8% vs. 24.4%; OR, 5.00; 95% CI, 3.18–7.88; P<0.00001; I^2^ = 0%) and 1-year (55.1% vs. 20.8%; OR, 7.40; 95% CI, 3.97–13.79; P<0.00001; I^2^ = 16%) follow-up periods. The results are summarized in Fig 3. Meta-regression models found no significant effects exerted by patient age (P = 0.166), gender (P = 0.939), LVEF (P = 0.226), AF duration (P = 0.138), hypertension (P = 0.252), or LAD (P = 0.145) upon sinus rhythm at discharge in Fig 4. Subgroup analysis of different ablation energy including cut and sew radiofrequency ablation, microwave and cryoablation demonstrated no significant difference affecting SR at discharge. Subgroup analysis according to the included number of patients or concomitant cardiac surgery type didn’t make a difference regarding SR at discharge. The results are summarized in Figs 5–7. Left atrial surgical ablation showed a good benefit in terms of freedom from atrial fibrillation at 1-year follow up (Hazard Ratio, 0.41, 95% CI, 0.37–0.46; P<0.00001; I^2^ = 92%; Fig 8). Meta-regression models found no significant effects exerted by patient age (P = 0.07), gender (P = 0.08), LVEF (P = 0.42), AF duration (P = 0.14), or LAD (P = 0.31) upon freedom form atrial fibrillation at 1 year follow up in Fig 9.

### Assessment of safety

#### Mortality

**Fig 10:**
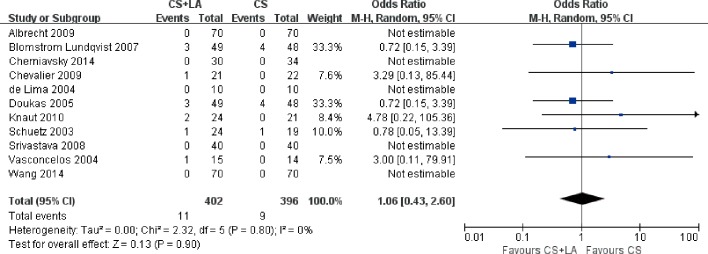
Forest plot of 30-day mortality, showing summary ORs with 95% confidence intervals for included studies.

**Fig 11:**
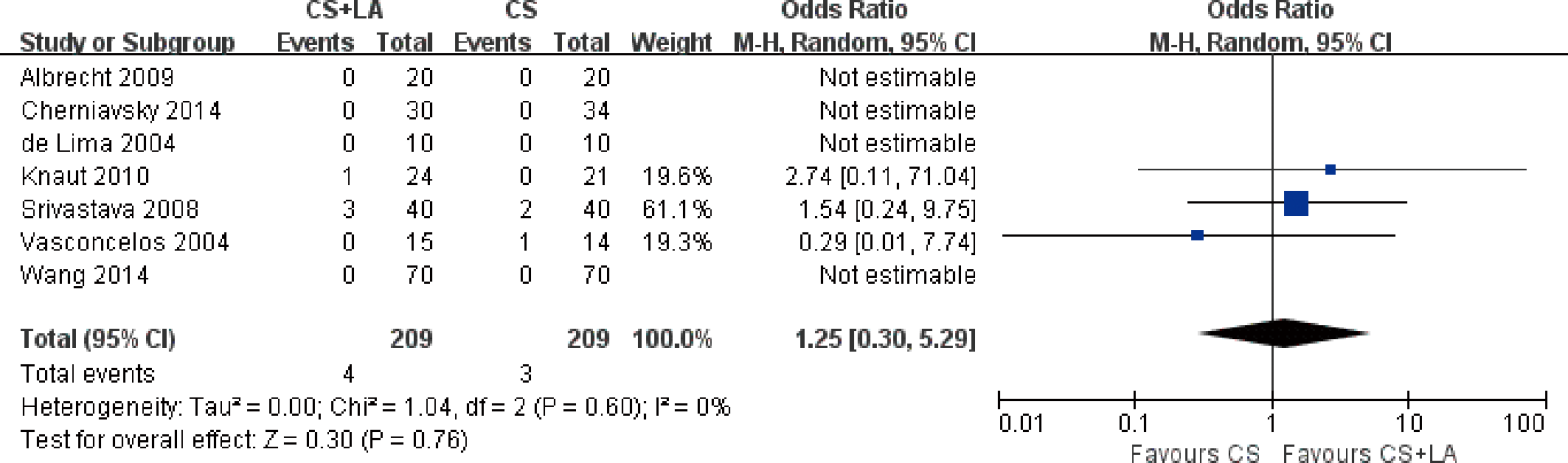
Forest plot of late mortality, showing summary ORs with 95% confidence intervals for included studies.

Mortality outcomes at 30 days were reported in all studies. The risk of 30-day all-cause mortality was not significantly different between CS + LA and CS groups at 30 days (2.7% vs. 2.3%; OR, 1.06; 95% CI, 0.43–2.60; P = 0.90; I^2^ = 0%; Fig 10). Furthermore, all-cause late mortality was also not significantly different (1.7% vs. 2.4%; OR, 1.25; 95% CI, 0.30–5.29; P = 0.76; I^2^ = 0%; Fig 11). No significant heterogeneity was observed in these two comparisons.

#### Neurological events

**Fig 12:**
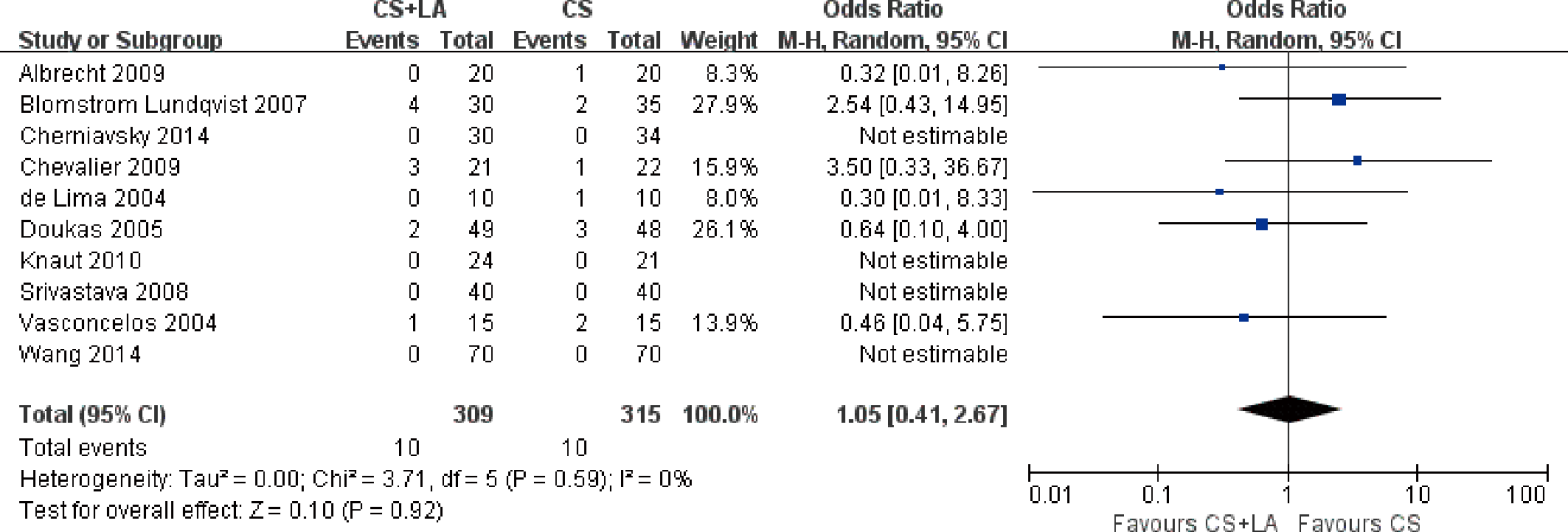
Forest plot of neurological events, showing summary ORs with 95% confidence intervals for included studies.

All but one study reported outcomes for neurological events. The rates of neurological events ranged from 0 to 10.3% in the studies. 4 of 10 studies with a total of 329 patients reported no neurological events. 3 of 10 studies with a total of 137 patients reported a rate of neurological events above 9%. Overall there was a comparable results between CS + LA and CS groups with no significant heterogeneity (3.2% vs. 3.2%; OR, 1.05; 95% CI, 0.41–2.67; P = 0.92; I^2^ = 0%; Fig 12).

#### Permanent pacemaker implantations

**Fig 13:**
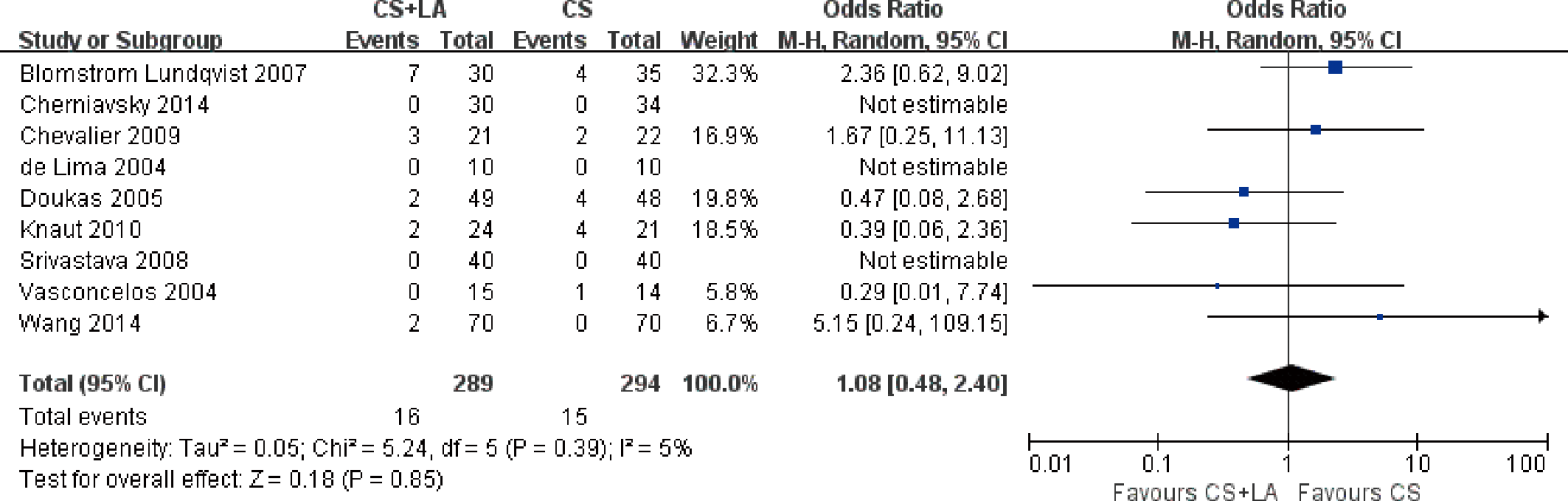
Forest plot of permanent pacemaker implantations, showing summary ORs with 95% confidence intervals for included studies.

Permanent pacemaker implantations were reported in nine out of eleven included studies. The permanent pacemaker implantation percentage was 4.7% on average in these studies with a range from 0 to 16.9%. 3 of 9 studies reported no permanent pacemaker implantation with 164 patients included. 3 of 9 studies reported a percentage over 11% with a total of 153 patients. Overall, there was no difference in pacemaker implantations whether left atrial ablation was performed or not (5.5% vs. 5.1%; OR, 1.08; 95% CI, 0.48–2.40; P = 0.85; I^2^ = 5%; Fig 13).

#### Reoperation for bleeding

**Fig 14:**
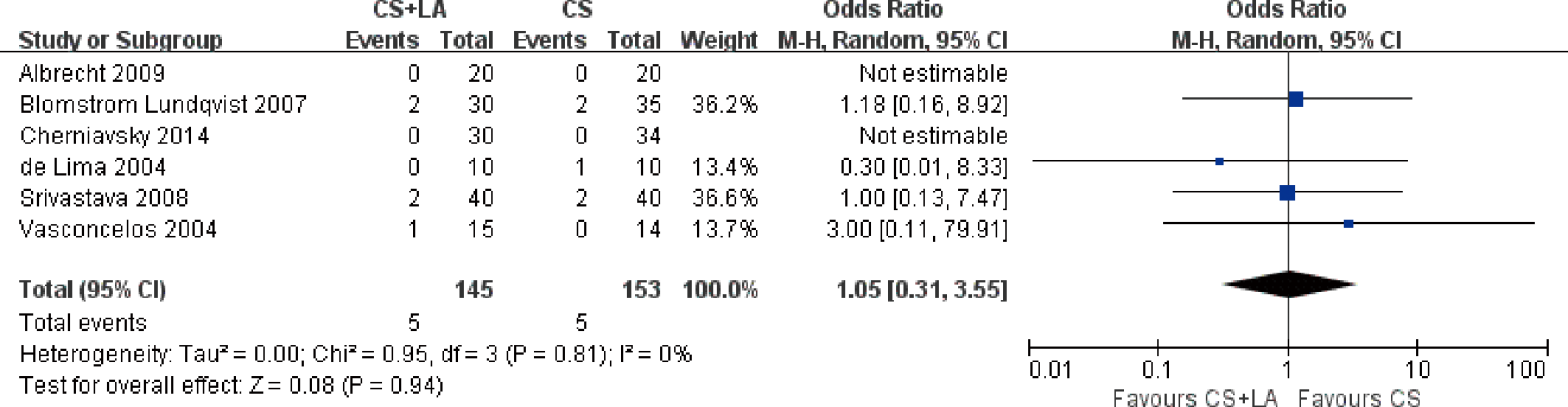
Forest plot of reoperation for bleeding, showing summary ORs with 95% confidence intervals for included studies.

Reoperation for bleeding were reported in six out of eleven included studies. The average reoperation for bleeding rate was 1.5%, ranging from 0 to 6.2%. 2 of 6 studies reported no reoperation for bleeding with a total of 104 patients. Overall, there was no difference in reoperation for bleeding between CS + LA and CS groups (5.3% vs. 5.1%; OR, 1.05; 95% CI, 0.31–3.55; P = 0.94; I^2^ = 0%; Fig 14).

### Publication bias

Egger’s test detected no publication bias for the major outcomes of this meta-analysis: 30 days mortality (P = 0.52), permanent pacemaker implantations (P = 0.23), neurological events (P = 73), and reoperations for bleeding (P = 0.22). Publication bias was found regarding sinus rhythm prevalence at 1 year (P = 0.02). After excluding the study of Chevalier or Wang, no publication bias was detected with P value of 0.09 and 0.06, respectively.

## Discussion

AF has been supposed to be caused by the multiple and disorganized re-entrant circuits in atrial walls. Cox-Maze III procedure firstly applied by Dr James L Cox in 1992 was the gold standard treatment for AF. During the past two decades, modified Cox-Maze procedures and multiple ablation energy improved the outcomes of surgical ablation compared with traditional Cox-Maze III procedure. A recent meta-analysis has confirmed the cardiac surgery with concomitant surgical ablation is an effective and safe strategy for AF[10]. However, the efficacy and safety of simplified left atrial surgical ablation were not established. Thus, our study aims to report the clinical outcomes of CS+LA versus CS through a meta-analysis of RCTs.

AF has been consistently proved to be an independent predictor of life and survival, which suggests that the restoration of SR in AF patients is a critical therapeutic strategy[24–27]. For example, the AFFIRM study proved that the prevalence of SR was an important, independent predictor of survival, after adjustment for clinical variables such as age and comorbidities[26]. The risk to die among patients in SR was almost half of those who did not improve from AF (adjusted hazard ratio, 0.53; 99% CI, 0.39 to 0.72; P<0.0001). In the present meta-analysis, we demonstrated that a higher SR prevalence in the CS+LA group at discharge (65.8% vs. 30.0%), 6-month (55.8% vs. 24.4%) and 1-year (55.1% vs. 20.8%) follow-up compared to CS group. Surgical ablation also improved the freedom from atrial fibrillation at 1-year follow up (HR, 0.41, 95% CI, 0.37–0.46; P<0.00001; I^2^ = 92). Previous meta-analyses had proved that concomitant surgical ablation was a safe and effective at restoring rhythm during cardiac surgery with no exception for mitral valve surgery[10, 11]. The surgical ablation group had a higher prevalence of sinus rhythm at all ≥ 12 month follow up (OR, 6.72; 95% CI 4.88 to 9.25; P<0.00001). The mortality, pacemaker implantation and neurological events were similar compared with no surgical ablation group. Another meta-analysis comparing biatrial ablation (BA) and left atrial ablation for AF found that the superiority of BA for restoring SR only maintained in 1 year with no difference beyond 1 year[28]. The SR prevalence in BA and LA group was similar for patients with follow-up beyond 1 year (59 vs 64%; OR 1.03; 95% CI 0.70–1.51; P = 0.87; I^2^ = 26%). Our meta-analysis proved that left atrial ablation was an effective therapeutic strategy to restore SR in persistent or permanent AF patients, which was a good supplement to the previous studies.

The present meta-analysis found acceptable 30-day mortality (range: 0–8.3%) and all-cause late mortality rates (range: 0–7.5%) in included RCTs with no significant difference between CS+LA and CS group. Considering five of eleven studies underwent the traditional cut-and-sew techniques, the low mortality justified the safety of cut-and-sew in left atrial maze surgery. However, the small number of patients in RCTs suggested this result should be treated with caution.

Performing surgical atrial fibrillation in conjunction with cardiac surgery was supposed to increase the risk for postoperative permanent pacemaker requirement[29]. The adjusted odds of permanent pacemaker implantation was higher in surgical ablation group than that in patients with no history of AF who underwent cardiac surgery (OR 2.7; 95% CI 1.7–4.4). In the present meta-analysis, permanent pacemaker implantations were similar in CS+LA and CS group with a proportion of 5.5% and 5.1%, which was lower than the 5.94 to 7.10% reported by a large retrospective study of the Society of Thoracic Surgeons National Cardiac Database[30]. This result suggested that LA lesion set was a safe procedure in terms of pacemaker implantation. It was consistent with a recent retrospective study which found that not left atrial but biatrial lesion set was the only statistically significant predictor of permanent pacemaker implantation after concomitant surgical ablation for AF through a univariate and multivariate analysis of 594 patients[31]. Demographic data, type of surgical procedure, and type of energy source did not have a significant impact of pacemaker implantation rate. The enhanced SR outcomes may reduce the incidence of permanent pacemaker implantation. However, this speculation should be taken with caution as studies reported different indications for pacemaker implantations.

No significant difference was found between CS+LA and CS group in terms of neurological events. Previous studies reported a protective effect from neurological events after Cox-Maze technique in AF patients undergoing concomitant cardiac surgery[32–35]. The negative outcome in our meta-analysis may be as a result of mixed factors including prosthetic valve implantation, oral anti-coagulant or anti-platelet drugs, and the interaction between antiarrhythmic and anti-coagulant drugs. We also found a similar reoperation rates for bleeding in these two groups. This result was logically reasonable as the reoperation for bleeding was similar in mixed BA and LA surgical ablation compared with cardiac surgery alone[10].

There are several limitations to our meta-analysis. Firstly, the RCTs included in this meta-analysis were small sample size studies. Secondly, the variant ablation or cut-and-sew lines in left atrial among RCTs may had influence on the efficacy of surgical ablation. Thirdly, the follow-up results beyond 1 year were not reported in most RCTs which cast doubts on the long-term efficacy of surgical ablation. Fourthly, AF monitoring in RCTs were based on ECG or 24h Holter at specific time point which may not be effective to detect the paroxysmal or asymptomatic recurrent episodes. Fifthly, publication bias was found in the present meta-analysis. However, the main outcomes were robust in sensitive analysis.

## Conclusion

We draw a conclusion that concomitant left atrial surgical ablation and cardiac surgery for persistent or permanent AF has a good efficacy for restoration of SR in 1 year following surgery. The 30 day mortality, late all-cause mortality, neurological events and permanent pacemaker implantation are of no significant difference between two groups. Thus, our meta-analysis demonstrates that left atrial surgical ablation is an effective therapeutic strategy for AF patients undergoing concomitant cardiac surgery without increased risk of mortality and morbidity.

## Supporting information

S1 File PRISMA checklist. (DOC)
